# RfGNBP5 Negatively Regulates Innate Immunity of Red Palm Weevil, *Rhynchophorus ferrugineus*, Against Distinctive Pathogens

**DOI:** 10.3390/microorganisms14071474

**Published:** 2026-07-05

**Authors:** Qun Deng, Bing Ma, Liwei Liu, Rong Hu, Waqar Sattar, Ziying Zhu, Xinghong Wang, Youming Hou, Zhanghong Shi

**Affiliations:** 1 State Key Laboratory of Agriculture and Forestry Biosecurity, Fujian Agriculture and Forestry University, Fuzhou 350002, China; dengqun6534@163.com (Q.D.); ma15534605636@163.com (B.M.); waqar.sattar917@gmail.com (W.S.); ymhou@fafu.edu.cn (Y.H.); 2 College of Plant Protection, Fujian Agriculture and Forestry University, Fuzhou 350002, China

**Keywords:** *Rhynchophorus ferrugineus*, Gram-negative bacteria-binding protein, insect immunity, alien insect pest, biocontrol

## Abstract

Insects are continuously exposed to diverse microorganisms, including symbionts and pathogens, and rely on pattern-recognition receptors (PRRs) to initiate innate immune responses. Gram-negative bacteria-binding proteins (GNBPs) constitute an important class of the pivotal PRRs in insect immunity. However, whether GNBPs participate in immune responses against taxonomically distinct pathogens remains poorly understood. Red palm weevil (RPW), *Rhynchophorus ferrugineus*, is a notorious stem-boring insect pest that has caused huge economic loss worldwide. Our preliminary data indicated that the expression of *RfGNBP5* could be induced dramatically upon the challenge of non-entomopathogenic bacteria *Escherichia coli*, *Staphylococcus aureus* and entomopathogenic fungus *Beauveria bassiana*. Here, it has been found that RfGNBP5 only possesses two conserved domains, a signal peptide and an intact glycoside hydrolase 16 (GH16) domain, but without the canonical carbohydrate-binding module (CBM39), implying that it might mediate insect immunity via its hydrolytic activity. RT-qPCR revealed that *RfGNBP5* was expressed at the highest level in the hemolymph compared with other tissues and was significantly upregulated after exposure to pathogens, such as *Escherichia coli*, *Staphylococcus aureus* and *Beauveria bassiana*. Furthermore, the knockdown of *RfGNBP5* potentiated the clearance efficiency of hemolymph against the invading pathogenic bacteria and fungi by increasing the expression level of two antimicrobial peptide genes, including *RfAttacin* in fat body and *RfColeoptericin* in gut, and augmenting the phenoloxidase (PO) activity in hemolymph. Taken together, these results suggested that RfGNBP5 acts as a negative regulator of RPW immunity, and it might be a potential candidate for further evaluation in RNAi-based pest management.

## 1. Introduction

The red palm weevil (RPW), *Rhynchophorus ferrugineus*, is a destructive pest of palm trees worldwide, and it has caused severe economic losses [1,2]. Use of chemical pesticides is the current major control strategy and the field investigations have found that RPW larvae have strong resistance against chlorpyrifos and imidacloprid [3]. Therefore, environmentally sustainable alternatives, particularly microbial biological control agents, have attracted increasing attention. Entomopathogenic nematodes such as *Steinernema* sp. and the entomopathogenic fungus, *Beauveria bassiana*, have shown promise for suppressing *R. ferrugineus* under laboratory and semi-field conditions. Unfortunately, economically feasible management with these biocontrol agents has not been realized at a significant scale under field conditions [4,5,6]. It is well-known that insect pests have mainly relied on their innate immunity against the attack of these biological agents. In battling these microbial infections, the pattern recognition receptors (PRRs) of insects serve as the pivotal sentries to discriminate the danger signals and further activate the immune responses [7,8,9]. In this context, deeply unveiling the function of these PRRs and their action mechanisms will facilitate the development of novel pest control methods by disturbing the finely tuned insect immunity.

Insects depend on innate immunity to defend against microbial invasion through various mechanisms. These include cellular responses such as phagocytosis, nodulation and encapsulation, as well as humoral responses that encompass antimicrobial peptide production, melanization and activation of the prophenoloxidase cascade [10,11]. The initiation and coordination of these immune responses are mediated by pattern-recognition receptors and immune mediators operating through conserved signaling pathways, particularly the Toll and IMD pathways [12,13]. Besides microbial pathogen-associated molecular patterns, endogenous damage-associated molecular patterns also play a role in immune activation. For instance, dorsal switch protein 1 (DSP1), an insect homolog of high mobility group box 1, has been found to activate Toll–Spätzle signaling and enhance immune responses, including phenoloxidase activity and antimicrobial peptide synthesis [14,15]. Therefore, understanding how PRRs and immune mediators regulate insect immune homeostasis could facilitate the identification of new targets for pest management.

In natural environments, insects are frequently exposed to a variety of microorganisms, including viruses, pathogenic bacteria, fungi, and natural enemies such as parasitoids, whether in a simultaneous or sequential manner. In addition to resisting pathogenic infections, insects may also utilize some environmental microorganisms to adapt to the fluctuating external conditions, suggesting that insect immunity does not simply remove the invading pathogenic microbes but also maintains the homeostasis of insect–microbe symbiosis [16]. Because current reports on insect immunity were mainly from the investigations with the challenge of a single pathogenic species, it still remains unclear on the mechanisms underlying the battle of the insect immune system against simultaneous or sequential attack by a variety of pathogens. The precise fast detection and recognition of exogenous and endogenous signals by insect PRRs is pivotal to trigger the innate immune responses to combat infections [17,18,19]. From this perspective, we thought that there might be some PRRs with the function to discriminate the distinctive kinds of pathogens in insects. Several groups of PRRs, such as peptidoglycan recognition receptors (PGRPs), β-1,3-glucan recognition proteins (βGRPs), Gram-negative bacterium-binding proteins (GNBPs), C-type lectins (CTLs) and scavenger receptors (SRs), have been well defined in insects [11]. Among them, βGRPs and GNBPs belong to the same PRR family, which can recognize β-glucans [12,20].

Typical insect GNBP/βGRPs often possess a signal peptide, a tandem N-terminal carbohydrate-binding module (CBM, function as microbial recognition) and a glucanase-like (GH16, function as triggering downstream immune pathways) domain in the C-terminal [12,21]. It is increasingly evident that a typical GNBP may be involved in the activation of insect prophenoloxidase cascade and Toll pathway by binding to different types of ligands, including β-1,3-glucan, lipopolysaccharide (LPS), laminarin and lipoteichoic acid [22,23]. For instance, *Manduca* βGRP-1 binds with and agglutinates Gram-negative and Gram-positive bacteria [24]. In *Drosophila melanogaster*, GNBP3 can agglutinate fungal cells and induce melanization in a Toll-independent manner [25]. Our previous multiomics-based investigations revealed that the expression level of *RfGNBP5* in RPW larvae was markedly up-regulated after exposure to *Escherichia coli*, *Staphylococcus aureus* and *Beauveria bassiana*, suggesting that it might mediate the activation of immunity against different kinds of pathogenic microbes [26]. More interestingly, CBM, which can enhance the catalytic efficiency of the carbohydrate-active enzymes against soluble or insoluble substrates [27], was not found in RfGNBP5, implying that it might be involved in insect immunity via a different manner compared with typical GNBP/βGRPs. In this study, the function and potential action mechanisms of RfGNBP5 in regulating antimicrobial peptide (AMP) genes’ expression, PO level and pathogen clearance of RPW larvae were determined by RNA interference (RNAi) and RT-qPCR. Our results indicated that RfGNBP5 acts as a negative regulator of innate immunity in RPW, thereby expanding the current knowledge on the functions of insect GNBP/βGRPs and highlighting a potential target for the RNAi-based control strategy by destroying insect immune homeostasis.

## 2. Materials and Methods

### 2.1. Insect Rearing

The experimental colonies of *Rhynchophorus ferrugineus* (RPW) were replenished by adults collected by sex pheromone-based traps (Zhangzhou Inger Co., Ltd., Zhangzhou, China) in the *Phoenix canariensis* plantation at Xialiao Village (25°07′ N, 118°21′ E), Nan’an City, Fujian Province. The adults were maintained at a 1:1 female-to-male ratio within ventilated transparent plastic containers (12 cm diameter, 10 cm height), and were fed with fresh sugarcane slices, which were replaced every 4–5 days. During the dissection of the discarded sugarcane slices, eggs were collected and transferred into clean glass Petri dishes (9 cm diameter) lined with soaked filter paper by a moistened soft-bristle brush. These dishes, along with the larvae, were then incubated in a climate chamber (Saifu ZRX-260, Ningbo Experimental Instrument Co., Ltd., Ningbo, China) under the following conditions: continuous darkness (L:D = 0:24), 27 ± 1 °C, and 75 ± 5% relative humidity (RH). The fourth instar larvae were used in the subsequent experiments.

### 2.2. Characterization of RfGNBPs

The open RPW genomic data were initially downloaded from the following link (https://www.ncbi.nlm.nih.gov/assembly/GCA_014462685.1/, accessed on 15 January 2026) [28]. *RfGNBP* genes were identified by running Local Blast-blastp by using DmGNBPs as the baits, with results accepted when the E value was less than 0.1. The cDNA sequence of *RfGNBPs* was translated by running ExPASY tools (http://www.expasy.org/) and the conserved functional domains were predicted by SMART (https://smart.embl-heidelberg.de/index2.cgi, accessed on 15 January 2026). Multiple sequence alignments of RfGNBPs with other insect GNBPs were completed and visualized by using MEGA 12.0 (Pennsylvania State University, University Park, PA, USA) and Jalview2.11.5.1 (University of Dundee, Dundee, UK), respectively. For phylogenetic analysis, 27 GNBP homologs from other insect species were selected based on BLASTP (NCBI, Bethesda, MD, USA) similarity and taxonomic representation across major insect orders, and a maximum-likelihood tree was constructed by running MEGA 12.0.

### 2.3. Putative Ligand Binding Assay of RfGNBP5

To investigate the interaction between RfGNBP5 and its potential ligands, the three-dimensional structure of RfGNBP5 was predicted using AlphaFold Server (https://alphafoldserver.com/). Subsequently, molecular docking assays on RfGNBP5 with different ligands, including LPS inner core oligosaccharide, glucosamyl muramyl pentapeptide (GMPP), a complete peptidoglycan (PGN) unit and β-1,3-glucan, were performed with AutoDock Vina 1.5.7 (The Scripps Research Institute, La Jolla, CA, USA). Detailed docking parameters were provided in Table S2. The three-dimensional structure of RfGNBP5 and the molecular docking results were visualized using PyMOL 3.1.0 (Schrödinger, LLC, New York, NY, USA) and Discovery Studio Visualizer 25. 1.0.24284 (Dassault Systèmes BIOVIA, San Diego, CA, USA).

### 2.4. Detection of RfGNBP5 Expression Level in Different Tissues of RPW Larvae

To determine the profiling of RfGNBP5 across tissues, including head, fat body, integument, foregut and mid/hindgut, the 4th instar larvae were surface sterilized with 75% ethanol followed by ddH_2_O rinsing, anesthetized on ice, and dissected by sterilized forceps in a laminar flow hood (Antai Airtech Co., Ltd., Suzhou, China). The tissues of three larvae were pooled into a 1.5 mL EP tube containing sterile PBS as a replicate, ensuring that each tissue comprised at least three biological replicates. Total RNA was extracted using Eastep^®^ Super Total RNA Extraction Kit (Promega Biotech Co., Ltd., Shanghai, China) according to the manufacturer’s instructions. Hemolymph total RNA was extracted using a hemolymph-specific RNA extraction kit (Aidlab Biotechnologies Co., Ltd., Beijing, China). The purity and concentration of extracted total RNA were evaluated using agarose gel electrophoresis and a ND2000 spectrophotometer (Thermo Fisher Scientific, Waltham, MA, USA), respectively. All samples were stored at −80 °C for further use. The first cDNA strand was synthesized using the FastKing gDNA Dispelling RT SuperMix (Tiangen Biotech Co., Ltd., Shanghai, China). The reaction system was constructed as follows: 4 µL of 5× FasKing-RT SuperMix, 1 µg of total RNA, and RNase-free water added to 20 µL.

For RT-qPCR experiments, reagents from 2× FastStart Universal SYBR Green Master (Roche, Basel, Switzerland) and the ABI 7500 Quantitative PCR System (Life Technologies, Carlsbad, CA, USA) were utilized. The reaction system was set up as follows: 10 µL of SYBR mix, 0.4 µL of Forward primer, 0.4 µL of Reverse primer, 1 µL of cDNA, and 8.2 µL of RNase-free water. The reaction program was set as follows: pre-denaturation at 95 °C for 10 min; denaturation at 95 °C for 15 s; annealing at 60 °C for 1 min; 40 cycles. *RfActin* was used as an internal control to calculate the gene’s relative expression level using the 2^−∆∆Ct^ method.

### 2.5. The Response Pattern of RfGNBP5 upon the Challenge of Pathogenic Microbes

To investigate the role of *RfGNBP5* in the immunity of RPW larvae against pathogenic infections, the 4th instar larvae were challenged with three different pathogens, containing Gram-negative bacterium *Escherichia coli*, Gram-positive bacterium *Staphylococcus aureus* and entomopathogenic fungus *B. bassiana*. Bacterial suspensions were prepared as described by Gong et al. [19]. Briefly, *E. coli* and *S. aureus* were cultured to logarithmic phase in LB medium at 37 °C while shaking at 200 rpm. The bacterial cells were harvested by centrifugation (8 min, 5000 g, 4 °C), washed three times with sterile PBS, and resuspended to a final OD_600_ of 1.6. For *B. bassiana*, a fresh spore suspension (2 × 10^6^ spores/mL) was prepared by harvesting spores from Potato Dextrose Agar (PDA) plates and filtering through sterile gauze to remove hyphal debris. A 1 µL aliquot of the bacterial or fungal suspension was injected into the body cavity using a 10 µL micro syringe (Gaoge Co., Ltd., Shanghai, China). The fat bodies of RPW larvae were collected at 24 h post-infection for total RNA extraction. Each treatment included three biological replicates, with the fat bodies of three larvae pooled for each replicate.

### 2.6. Effect of RfGNBP5 Silencing on the Phenoloxidase Activity and Pathogen Clearance Ability in Hemolymph of RPW Larvae

*dsRfGNBP5* and dseGFP were synthesized according to the methods described by Dawadi et al. with MEGAscript^®^ RNAi Kit (Thermo Fisher Scientific, USA). A total of 1 μL of 1000 ng/µL dsRNA was injected into the hemocoel of fourth-instar larvae. The guts and fat bodies were then collected through dissection 48 h after the delivery of dsRNA. Each experimental group included three replicates, with three larvae pooled per replicate.

To detect the effect of *RfGNBP5* silencing on the pathogen-clearing ability of RPW larvae hemolymph, 1 μL of each pathogen suspension (approximately 8 × 10^5^ *E. coli* cells, 6.4 × 10^5^ *S. aureus* cells and 2 × 10^3^ spores *B. bassiana*) was injected into the body cavity of larvae at 45 h after dsRfGNBP5 injection, respectively. Hemolymph (150 μL) was collected at 3 h post-injection. In bacterial infection assays, because both eGFP-tagged *E. coli* DH5α and *S. aureus* contained the ampicillin-resistance gene (*AmpR*), 100 μL hemolymph was spread on the LB agar medium with ampicillin and incubated for 16 h at 37 °C. The number of recovered bacterial colonies was counted under a Nikon ECLIPSE NI stereomicroscope (Nikon Corporation, Tokyo, Japan). For the assays on the challenge of fungus, 1 μL hemolymph was loaded onto a hemocytometer sterilized with 75% ethanol, and the number of recovered fungal spores was counted using a Nikon ECLIPSE NI microscope under the condition of differential interference contrast (DIC).

To determine the effect of *RfGNBP5* knockdown on the phenoloxidase (PO) level, hemolymph was collected from RPW larvae at 48 h post *dsRfGNBP5* injection, and PO level was quantified with an Insect PO ELISA Kit (Sinobestbio Co., Ltd., Shanghai, China). Following the kit’s protocol, 50 μL standard solutions (0, 3, 6, 12, 24, 48 U/mg) and 1:10 diluted hemolymph samples were loaded into a cell of a 96-well microplate, respectively. Then, 60 min after incubation with horseradish peroxidase (HRP)-conjugated detection antibody at 37 °C, the plate was washed five times. Chromogenic reaction was initiated by sequentially adding 50 μL of substrates A and B to each well, and the plate was maintained at 37 °C in the dark for 15 min before terminating the reactions. The absorbance value of each well was measured at 450 nm using a CMax Plus microplate reader (Molecular Devices, LLC, Shanghai, China).

### 2.7. Data Analysis and Processing

One-way analysis of variance (ANOVA) was utilized to assess the significance in the expression level of *RfGNBP5* across different tissues and under the challenge of a variety of pathogenic microbes. A Student’s *t*-test was run to determine the significance between the two groups. A *p*-value < 0.05 was considered statistically significant and all results were presented as mean ± standard deviation (SD). All statistical analyses were carried out using IBM SPSS 21.0 (IBM Corp., Armonk, NY, USA), and all figures were generated with GraphPad Prism 10.1.2 (GraphPad Software, Boston, MA, USA).

## 3. Results

### 3.1. Sequence Analysis and the Potential Function of RfGNBPs

**Figure 1:**
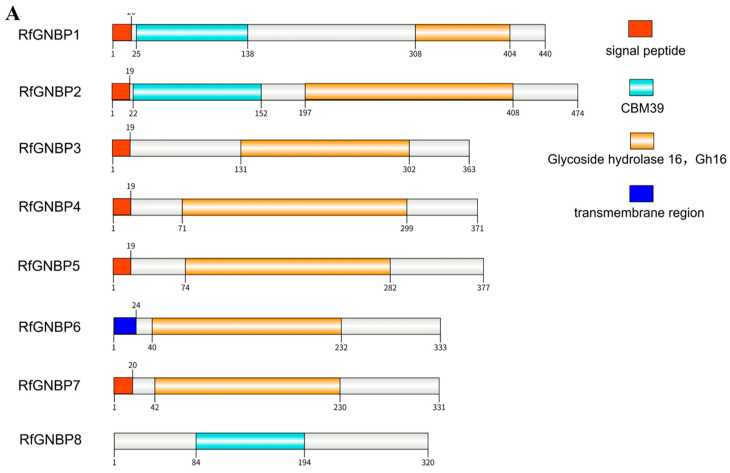

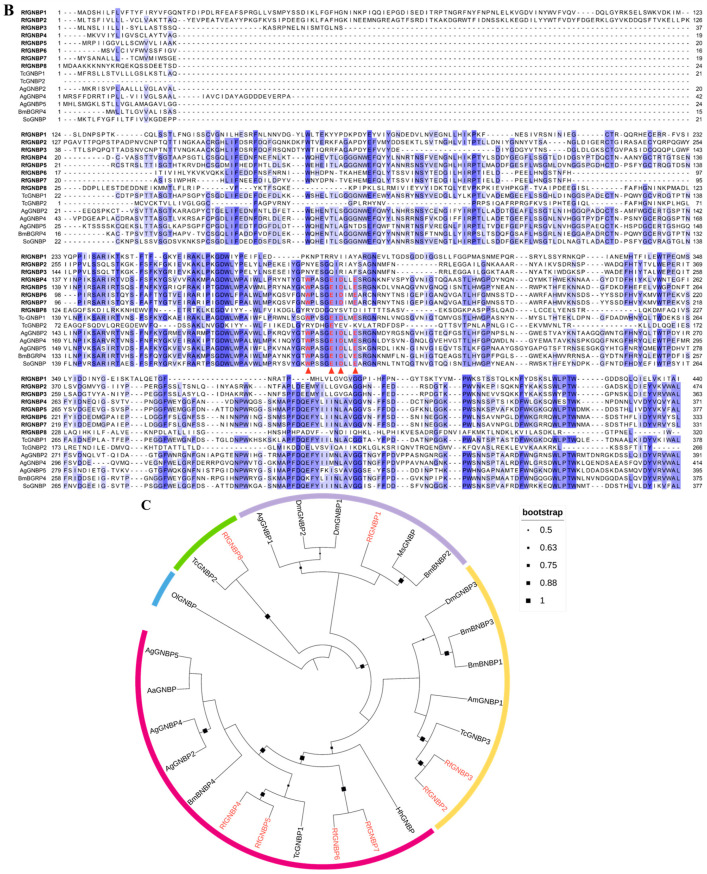
Characterization and phylogenetic relationship of RfGNBPs with other insect GNBP/βGRPs. (**A**) Domain architecture of RfGNBPs, such as a signal peptide (red), a CBM39 domain (sky blue) and a GH16 domain (orange). Domain length (amino acid residues) was indicated by the numbers below. (**B**) Multiple alignments of the amino acid sequences of RfGNBPs with other insect GNBPs. Four key residues containing Trp (W), Glu (E), Asp (D) and Glu (E) were highlighted in red triangles. (**C**) Phylogenetic relationship of RfGNBPs with other insect GNBPs. The distinctive color indicated that these RfGNBPs were assigned into different clade. The accession numbers of other insect GNBP/βGRPs in GeneBank are shown in Table S2.

Eight GNBP genes were identified in the available RPW genome. RfGNBP1 and RfGNBP2 consist of a signal peptide, a CBM39 domain and a GH16 domain. In contrast, six other RfGNBPs are classified as noncanonical PRRs. For instance, five RPW GNBPs, including RfGNBP3, RfGNBP4, RfGNBP5, RfGNBP6 and RfGNBP7, comprise a signal peptide and a GH16 domain. While RfGNBP-8 only has a CBM39 domain (Figure 1A). Multiple sequence alignments revealed that RfGNBP-4, RfGNBP5, RfGNBP6 and RfGNBP7 contain four conserved amino acid residues, including Trp (W), Glu (E), Asp (D) and Glu (E), being required for the glycoside hydrolase activity (Figure 1B). Phylogenetic analysis showed that eight RfGNBPs were assigned into four branches (Figure 1C), implying that they could mediate the immune responses of *R. ferrugineus* in two different ways, such as detecting the pathogens alone or in cooperation with other PRRs and forming the “attack complexes” with other immune effectors [16]. For instance, RfGNBP1 was classified into the branch with DmGNBP1, suggesting that it might be involved in RPW immunity via the first way. Moreover, RfGNBP4, RfGNBP5, RfGNBP6 and RfGNBP8 were clustered into the same branch with TcGNBP1 and TcGNBP2, respectively. These results implied that these RfGNBPs play a critical role in the immune response of this pest against various pathogenic microbes [29,30].

### 3.2. Putative Ligand-Binding Assay of RfGNBP5

**Figure 2:**
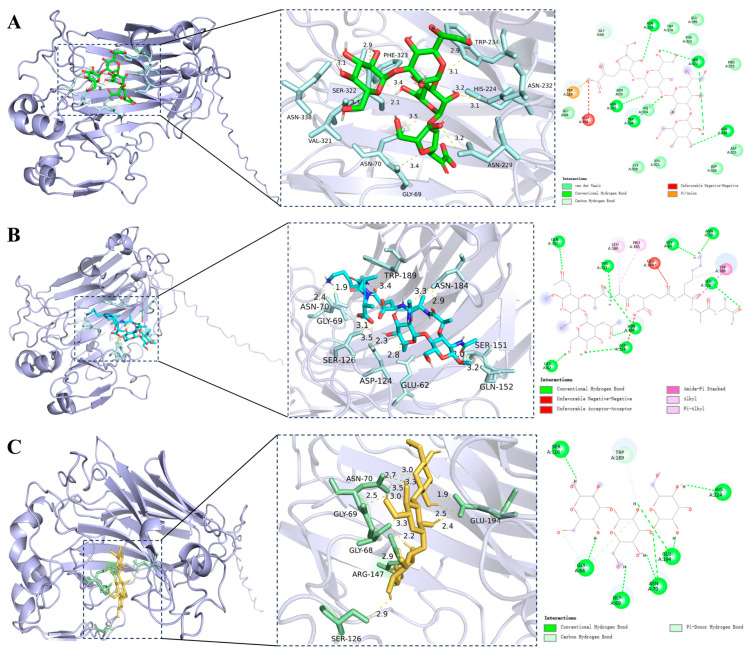
Molecular docking analysis on the binding capability of RfGNBP5 to three carbohydrate ligands, including the core chemical group of lipopolysaccharide (LPS) oligosaccharide (**A**), peptidoglycan (PGN) (**B**) and β-1,3-glucan (**C**). LPS, PGN, or β-1,3-glucan were represented in green, blue and yellow, respectively.

The open reading frame (ORF) length of *RfGNBP5* is 1134 base pairs (bps), encoding a protein consisting of 377 amino acids with an estimated molecular mass of 42.59 kD and an isoelectric point of 5.69. Molecular docking results suggested that LPS, PGN and β-1,3-glucan may interact with RfGNBP5 through several candidate residues. For example, the core chemical group of LPS, oligosaccharide, was predicted to bind to ASN229, ASN232, TRP234, SER322 and ASN338, forming multiple canonical hydrogen bonds with SER322 and π-π stacking mediated by TRP234 (Figure 2A). PGN may interact with GLU62, GLY69, ASN70, ASP124, SER126, GLN152, ASN184 and TYR311 through synergistic hydrogen bonding, amide-π stacking and hydrophobic forces (Figure 2B). In the case of β-1,3-glucan (a trisaccharide structure), the docking analysis suggested it might interact with GLY68, GLY69, ASN70, SER126, HIS224 and GLU194 by ASN70/SER126-driven hydrogen bonds (Figure 2C). In addition to hydrogen-bonding, van der Waals forces, carbon–hydrogen bonds and π-donor hydrogen bonds were identified in the complexes, with local unfavorable charge repulsion in LPS/PGN systems reflecting conformational plasticity. Notably, our docking results suggested that GLY69, ASN70 and SER126 were the essential conserved amino acid residues for the broad-spectrum binding activity of GNBPs to PAMPs, with GLY69 and ASN70 engaging all three tested ligands, and SER126 participating in PGN and β-1,3-glucan recognition.

### 3.3. The Expression Profile of RfGNBP5 Across Different Tissues and Its Response to the Attack from a Variety of Pathogenic Microbes

**Figure 3:**
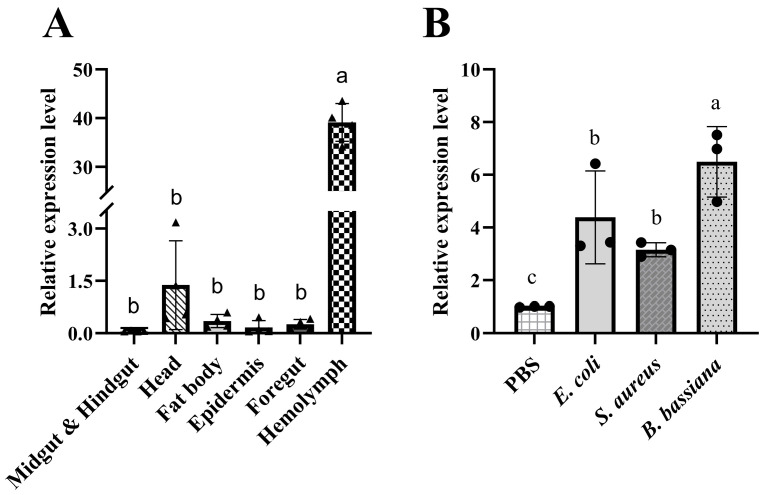
Expression analysis of *RfGNBP5* in RPW larvae. (**A**) The expression profiling of *RfGNBP5* across different tissues. (**B**) The response of *RfGNBP5* to the challenge of various pathogenic microbes. Tissues from three larvae were pooled as one biological replicate, and three biological replicates were used for each tissue. The data was represented by mean ± SD. Different letters above the bars indicated significant differences among groups, as determined by one-way ANOVA followed by Tamhane’s T2 test (*p* < 0.05). Bars sharing the same letter are not significantly different.

RT-qPCR analysis revealed that *RfGNBP5* was expressed in multiple tissues of the RPW larvae, including the head, epidermis, fat body, foregut, midgut and hindgut and hemolymph. The significance of its expression across these different tissues was determined (ANOVA: *F*_5,12_ = 358.078, *p* < 0.001, Figure 3A). The highest expression level of RfGNBP5 was detected in the hemolymph, whereas no significant difference was observed among the other examined tissues, as indicated by the same letter in Figure 3A. Interestingly, the highest expression level of *RfGNBP5* was detected in the hemolymph. Furthermore, the challenge with *E. coli*, *S. aureus*, and *B. bassiana* could markedly induce the expression of *RfGNBP5* in hemolymph (ANOVA: *F*_3,8_ = 12.773, *p* < 0.01) (Figure 3B), suggesting that RfGNBP5 may play a pivotal role in the systemic immunity of this pest.

### 3.4. Effect of RfGNBP5 Silencing on the Immunocompetence of RPW Larvae

**Figure 4:**
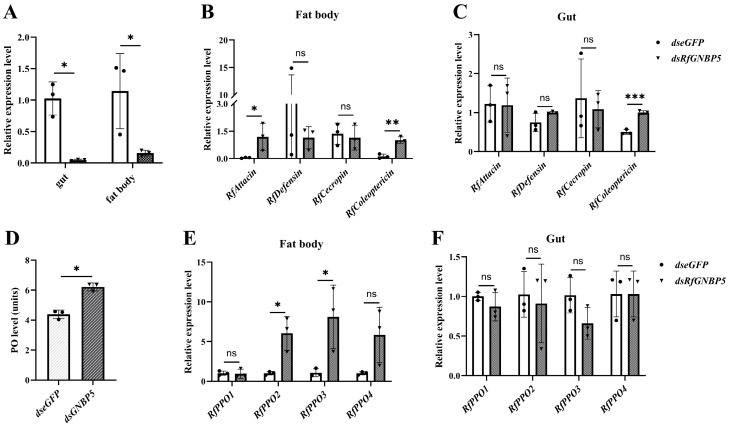
Effects of *RfGNBP5* silencing on the immunocompetence of RPW larvae. For *RfGNBP5* knockdown, the fourth-instar larvae were injected with *dsRfGNBP5* or *dseGFP*, and then their fat body, gut and hemolymph were collected at 48 h after dsRNA injection. (**A**) Silencing efficiency of *RfGNBP5* in fat body and gut. Effect of *RfGNBP5* silencing on the expression level of antimicrobial peptide genes (**B**,**C**), PO activity in hemolymp (**D**) and prophenoloxidase genes (**E**,**F**). The data were presented as mean ± SD from three biological replicates. Significant differences between the *dsRfGNBP5* and *dseGFP* groups were determined using Student’s *t*-test. “*” on the histograms indicated that the significance was determined between two groups (*: *p* < 0.05, **: *p* < 0.01, ***: *p* < 0.001).

At 48 h after the delivery of ds*RfGNBP5*, the expression level of *RfGNBP5* was significantly reduced with the efficiency of 76.1% and 95.7% in fat body (*t*-test: *t* = 2.844, *df* = 4, *p* < 0.05) and gut (*t*-test: *t* = 6.462, *df* = 4, *p* < 0.01), respectively (Figure 4A). To determine the impact of *RfGNBP5* knockdown on the immunocompetence, the expression level of four AMPs (*RfAttacin*, *RfDefensin*, *RfCecropin* and *RfColeoptericin*), *RfPPOs* and PO level in hemolymph were compared between the two groups. Our data indicated that the knockdown of *RfGNBP5* dramatically upregulated the relative expression level of *RfAttacin* (*t*-test: *t* = −2.661, *df* = 4, *p* < 0.05), *RfColeoptericin* (*t*-test: *t* = −7.291, *df* = 4, *p* < 0.01), *RfPPO2* (*t*-test: *t* = −4.045, *df* = 4, *p* < 0.05) and *RfPPO3* (*t*-test: *t* = −3.019, *df* = 4, *p* < 0.05) in fat body and *RfColeoptericin* in gut (*t*-test: *t* = −10.879, *df* = 4, *p* < 0.001) (Figure 4B,C,E,F). Moreover, PO level in hemolymph of RPW larvae was also increased by 35% compared to the controls (*t*-test: *t* = −8.142, *df* = 4, *p* < 0.01) (Figure 4D). These results indicated that silencing *RfGNBP5* significantly enhanced the expression of immune effector genes and PO level, suggesting that *RfGNBP5* may function as a negative regulator of immune response in *R. ferrugineus*.

### 3.5. Effect of RfGNBP5 Silencing on the Pathogen Clearance in Hemolymph of RPW Larvae

**Figure 5:**
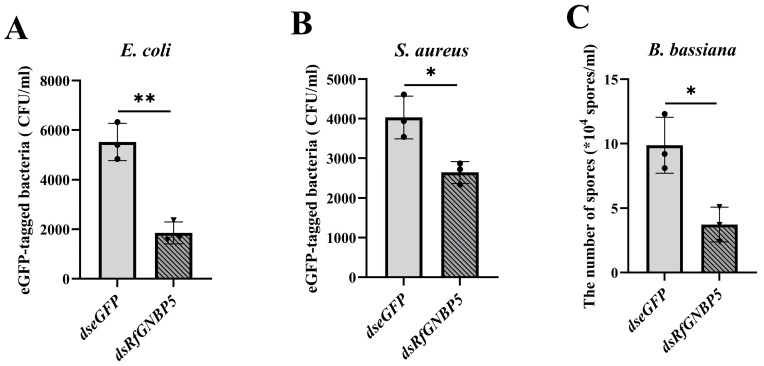
The effect of *RfGNBP5* silencing on the ability of RPW larvae to eliminate *Escherichia coli* (**A**), *Staphylococcus aureus* (**B**) and *Beauveria bassiana* (**C**). At 45 h after dsRNA injection, larvae were challenged with *E. coli*, *S. aureus*, or *B. bassiana*, and hemolymph was collected at 3 h post-infection to evaluate pathogen clearance. The data were presented as mean ± SD from three biological replicates. Significant differences between the *dsRfGNBP5* and *dseGFP* groups were determined using Student’s *t*-test. “*” indicated that the significance was detected between two groups (*: *p* < 0.05, **: *p* < 0.01).

Our findings revealed that the number of *E. coli* (1853.33 ± 440.61 CFU/mL) and *S. aureus* (2643.33 ± 273.19 CFU/mL) CFUs recovered from the hemolymph of *RfGNBP5*-silenced RPW larvae were significantly lower than those found in the controls insects (*t*-test for *E. coli*: *t* = 7.281, *df* = 4, *p* < 0.01, *t*-test for *S. aureus*: *t* = 3.965, *df* = 4, *p* < 0.05, Figure 5A,B). Similarly, the knockdown of *RfGNBP5* significantly improved the clearance ability of this pest to remove the invading fungal species in hemolymph (*t*-test: *t* = 4.146, *df* = 4, *p* < 0.05, Figure 5C). Consequently, these results confirmed that *RfGNBP5* silencing enhanced the pathogen clearance ability of RPW larvae, supporting that RfGNBP5 serves as a negative regulator in the immune responses of this pest.

### 3.6. Effect of RfGNBP5 Silencing on the Expression Level of Antimicrobial Peptide Genes in Fat Body of RPW Larvae Under Exposure to Pathogens

**Figure 6:**
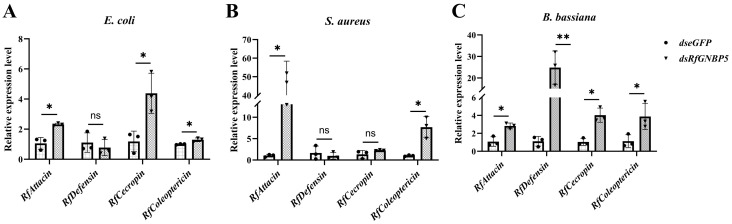
The effect of *RfGNBP5* silencing on the expression level of antimicrobial peptide genes in fat body of RPW larvae under the challenge of *Escherichia coli* (**A**), *Staphylococcus aureus* (**B**) and *Beauveria bassiana* (**C**). At 45 h after dsRNA injection, larvae were challenged with *E. coli*, *S. aureus*, and *B. bassiana*, and fat body were collected at 48 h after dsRNA injection. The data were presented as mean ± SD from three biological replicates. Significance between the *dsRfGNBP5* and *dseGFP* group were determined using Student’s *t*-test. “ns” presented that no significance was found between two groups, and “*” indicated that significance was determined between two groups (*: *p* < 0.05, **: *p* < 0.01).

To further clarify the mechanisms underlying the effect of *RfGNBP5* silencing on the pathogen clearance ability of RPW larvae, RT-qPCR was used to determine the changes in the expression level of AMP genes in the fat body of RPW larvae following pathogen challenge. Upon the challenge of *E. coli*, the higher expression level of *RfAttacin* (*t*-test: *t* = −5.419, *df* = 4, *p* < 0.01) and *RfColeoptericin* (*t* = −3.948, *df* = 4, *p* < 0.01) were found in the fat body of *RfGNBP5*-silenced larvae (Figure 6A). The attack of *S. aureus* significantly increased the expression level of *RfAttacin* (*t*-test: *t* = −2.954, *df* = 4, *p* < 0.05) and *RfColeoptericin* (*t*-test: *t* = −6.643, *df* = 4, *p* < 0.05) in the fat body of *RfGNBP5*-silenced RPW larvae as well (Figure 6B). Under the exposure of *B. bassiana*, the expression level of *RfAttacin* (*t*-test: *t* = −4.849, *df* = 4, *p* < 0.05), *RfDefensin* (*t*-test: *t* = −5.302, *df* = 4, *p* < 0.01), *RfCecropin* (*t*-test: *t* = −5.859, *df* = 4, *p* < 0.01) and *RfColeoptericin* (*t*-test: *t* = −2.923, *df* = 4, *p* < 0.05) in fat body of *RfGNBP5*-silenced RPW larvae were markedly higher than those in the control group (Figure 6C). Therefore, these data indicated that *RfGNBP5* silencing leads to significant upregulation in the expression level of antimicrobial peptide genes in the fat body under the pathogenic challenge.

## 4. Discussion

In their living environments, insect pests are often likely under the sequential or simultaneous attack of a variety of pathogenic microbes such as bacteria, fungi and viruses. Precise fast detection and discrimination of the invading pathogens by PRRs is a primary and critical step for the activation of insect immunity to combat them. Certain GNBP/βGRPs, such as PxβGRP [31] and TcGNBP1 [29], have been found to show a broad binding activity towards Gram^+^, Gram^−^ bacteria and fungi, implying that these PRRs can initiate the acute activation of immunity in the context of evolutionary biology. Interestingly, a circulating PRR with a similar role, RfGNBP5, was characterized in a destructive stem-boring insect pest. RfGNBP5 possesses the same functional domains as TcGNBP1, comprising a signal peptide and a Glycoside hydrolase 16 (GH16) domain. However, knockdown of *RfGNBP5* resulted in the marked elevation of RPW larvae immunocompetence, and this was in sharp contrast to the function of *Tribolium castaneum* GNBPs [29,30]. To the best of our knowledge, RfGNBP5 is the first member of the GNBP/βGRP family to act as the negative regulator of insect immunity compared with the verified GNBPs from *D. melanogaster* and other Lepidoptera insects [11,12].

Like other germline-encoded PRRs, insect GNBP/βGRPs are able to sense the foreign microbes, containing bacteria and fungi, in hemolymph and mount the immune responses [12]. The GNBP/βGRP family members, being designated into two distinct subfamilies, the PRR and glucanase subfamily, have been widely identified from insect species of various orders across Diptera, Lepidoptera and Coleoptera [20]. It is well-known that these GNBP/βGRPs are often involved in insect immunity by stimulating the expression of AMP genes and activating the proPO cascades [29,30,31]. In the accessible RPW genome data [28], eight GNBP genes, which are twice that of two model insects, *D. melanogaster* and *T*. *castaneum,* were found, indicating that an obvious gene amplification has happened in this gene family of RPW. Typical insect GNBP/βGRPs are characterized by the presence of a signal peptide, a carbohydrate-binding module 39 (CBM39) in the N-terminal and a Glycoside hydrolase 16 (GH16) domain in the C-terminal [12]. In the current investigation, we found that two GNBPs of *R. ferrugineus*, RfGNBP1 and RfGNBP2, have the common structural arrangement as the typical insect ones. In contrast, RfGNBP8 only contains a carbohydrate-binding module and five other RfGNBPs just comprise a putative GH16 domain. The existence of these conserved functional domains implied that these RfGNBPs have important, diverse roles in the immunity of this notorious pest.

Our preliminary multiomics data revealed that the expression level of *RfGNBP5* was dramatically induced by the challenge of pathogenic bacteria and fungi [26]. Phylogenetic analysis showed that RfGNBP5 was clustered into the same clade with TcGNBP and assigned to the glucanase subfamily. TcGNBP GH16 has the ability to bind to glucan and LPS [29]. This characteristic suggests that RfGNBP5 might participate in the innate immunity of *R. ferrugineus* by recognizing different PAMPs, which was further supported by the highest expression level of *RfGNBP5* found in hemolymph. Additionally, the expression level of *RfGNBP5* in hemolymph could be significantly induced by the challenge of *E. coli*, *S. aureus* and *B. bassiana*. These findings suggested that RfGNBP5 plays a crucial role in mediating the immune responses of this pest against pathogenic infections.

Current evidence indicates that insect GNBP/βGRPs mainly contribute to their innate immune responses in two different ways. Firstly, some GNBP/βGRPs act as the typical PRRs to activate the Toll pathway and the proPO cascades by directly recognizing fungal β-1,3-glucans [32] or cooperating with PGRPs to recognize the lysine-type peptidoglycan [18,33,34]. Secondly, certain GNBP/βGRPs form the “attack complexes” with other immune effectors to promote the antifungal defenses [20]. Silencing of GNBP/βGRP genes with the function described above by in vivo RNA interference often impaired the immune defenses of insects against pathogens [29,30,31]. Surprisingly, our data demonstrated that the silencing of *RfGNBP5* led to potentiated immunity, characterized by significant upregulation of AMP genes, such as *RfAttacin* and *RfColeoptericin* in fat body, elevated PO level and stronger bacterial clearance ability in hemolymph, indicating that RfGNBP5 may function as a negative regulator of *R. ferrugineus* immune responses. This finding was in sharp contrast with the reported role of the typical insect GNBP/βGRPs [16,29,30]. In *T. castaneum*, RNAi knockdown of genes in the IMD pathway strongly impaired the expression of *Attacin* and *Coleoptericin* [13]. Here, we found that the elevated expression levels of *RfAttacin* and *RfColeoptericin* were elevated by *RfGNBP5* silencing, indicating that these two AMPs in beetles are primarily under the control of the IMD signaling pathway [35]. The presence of the GH16 domain in RfGNBP5 suggested that it might have hydrolase activity. As a result, RfGNBP5 may limit the availability of immunostimulatory microbial ligands in the hemolymph to avoid the induced expression of the downstream AMP genes. However, these speculations need further biochemical validations by employing recombinantly expressed wild-type and site-directed mutagenesis proteins.

It has been found that induced antimicrobial activity in hemolymph starts to increase only when 99.5% of the invading bacteria have been removed [36]. These results imply that induced antimicrobial compounds function primarily to protect the insect against the bacteria that persist within their body, rather than to clear microbial infections at the early stage of infection. Because of its potential hydrolase activity, we hypothesized that RfGNBP5 might serve as the pioneer protein to recruit other immune effectors, such as RfPGRP-LB and -S1 with amidase activity, by binding and degrading the carbohydrates in the microbial cell wall and forming a hub to combat pathogens at the initial challenge stage. The employment of immune responses is costly for the host [10]. In this context, we thought that RfGNBP5 might fight against the invading pathogens with its hydolytic activity in hemolymph as a surveillance PRR, and this action of RfGNBP5 without triggering downstream immune pathways can lower the cost of using insect immune effector systems.

## 5. Conclusions

In conclusion, this study unveiled a non-canonical member of the GNBP/βGRP family, RfGNBP5, that may function as a negative regulator of *R. ferrugineus* immunity. RNAi-mediated silencing of RfGNBP5 led to increased expression of AMP genes, enhanced PO activity and improved pathogen clearance, supporting its potential role in maintaining immune homeostasis in this pest. These findings expand the known functional spectrum of the GNBP/βGRPs family and suggest that RfGNBP5 may represent a promising candidate for further evaluation in dsRNA-based pest management. However, the precise biochemical mechanism underlying RfGNBP5-mediated immune regulation remains to be clarified in future studies.

## Data Availability

The data presented in this study are available on request from the corresponding authors.

## Supplementary Materials

The following supporting information can be downloaded at: https://www.mdpi.com/article/10.3390/microorganisms14071474/s1, Table S1: The primers were used in this study; Table S2: GenBank accession numbers of insect GNBP/βGRPs that were used for phylogenetic analysis; Table S3: The parameters were employed for molecular docking assays.
