# Supplementary material for: RfGNBP5 Negatively Regulates Innate Immunity of Red Palm Weevil, Rhynchophorus ferrugineus, Against Distinctive Pathogens
Source: Microorganisms. 2026 Jul 5;14(7):1474. doi: 10.3390/microorganisms14071474 (PMC13414360; doi:10.3390/microorganisms14071474)
Supplement: Supplementary file 1 [file microorganisms-14-01474-s001.zip › microorganisms-4385750-supplementary.pdf]

**Supplementary Materials**

Table S1 The primers were used in this study.

| Primers | Sequences (5’ - 3’) |
|---|---|
| RT-qPCR | |
| *RfActin* | SP: CCAAGGGAGCCAAGCAATT |
| | AP: CGCTGATGCCCCTATGTATGT |
| *RfGNBP5* | SP: CGACGAGGAGTCTAACGACG |
| | AP: CGCCAGCCAAGGTAATTTCG |
| *RfAttacin* | SP: TGGTTCTGGTGCCCAAGTGA |
| | AP: GCCATAACGATTCTTGTTGGAGTA |
| *RfDefensin* | SP: TTCGCCAAACTTATCCTCGTG |
| | AP: GGGTGCTTCGTTATCAACTTCC |
| *RfCecropin* | SP: CAGAAGCTGGTTGGTTGAAGA |
| | AP: GCAACACCGACATAACCCTGA |
| *RfColeoptericin* | SP: TCGTGGTTTCTACCATGTTCACT |
| | AP: TCAGCTAAAACCTGATCTTGGA |
| *RfPPO1* | SP: TAACGCGCGATGATTTGCTG |
| | AP: TCTCAACCTCCACCATTGGC |
| *RfPPO2* | SP: TTGCCATCTTGCACCGTAGT |
| | AP: TACCACGTTGGCTTCCTCAC |
| *RfPPO3* | SP: GTACAGGCCGTTCACGTACA |
| | AP: AGCCAGGGATTGCCTCTTTC |

| | |
|---|---|
| *RfPPO4* | SP: TTACGCGCTGTCTGTTGCTA |
| | AP: TAGGGATACGGGCACCTTGA |
| RNAi | |
| *dsRfGNBP5* | SP: taatacgactcactatagggATTACGCCAATAACCGTTCG |
| | AP: taatacgactcactatagggTTCGCTTCATTGTAGGGTCC |
| *dseGFP* | SP: taatacgactcactatagggCAGTGCTTCAGCCGCTAC |
| | AP: taatacgactcactatagggGTTCACCTGCCGTTCTTGA |

Table S2 GenBank accession numbers of insect GNBP/βGRPs were used for the phynogenetic analysis.

| Species) | Abbreviation | Accession No. |
|---|---|---|
| *Rhynchophorus ferrugineus* | RfGNBP1 | KAF7272955.1 |
| | RfGNBP2 | KAF7268732.1 |
| | RfGNBP3 | KAF7268731.1 |
| | RfGNBP4 | KAF7274037.1 |
| | RfGNBP5 | KAF7274036.1 |
| | RfGNBP6 | KAF7268734.1 |
| | RfGNBP7 | KAF7268733.1 |
| | RfGNBP8 | KAF7278011.1 |
| *Anopheles arabiensis* | GNBP | XP_040154907.1 |
| *Anopheles gambiae* | AgGNBP1 | XP_040227006.2 |
| | AgGNBP2 | XP_312116.4 |
| | AgGNBP4 | XP_040151612.1 |
| | AgGNBP5 | XP_313748.3 |
| *Apis mellifera* | AmGNBP1 | NP_001157186.1 |
| *Bombyx mori* | BmBNBP1 | NP_001036840.1 |
| | BmBNBP2 | NP_001037450.1 |
| | BmBNBP3 | NP_001128672.1 |
| | BmBNBP4 | XP_037874607.1 |
| *Halyomorpha halys* | GNBP | XP_014282534.1 |
| *Manduca sexta* | GNBP | XP_037294821.1 |
| *Sitophilus oryzae* | GNBP | XP_030761868.1 |
| *Tribolium castaneum* | TcGNBP1 | XP_970010.1 |
| | TcGNBP2 | NP_001164284.1 |
| | TcGNBP3 | XP_972063.1 |
| *Oryzias latipes* | GNBP | BAD93252.1 |

Table S3 The parameters that were employed for the molecular docking assays.

| Ligand | Receptor | Center x | Center y | Center z | Size x | Size y | Size z | Number of runs | Binding energies (kcal/mol) |
|---|---|---|---|---|---|---|---|---|---|
| Core_oligosaccharide | RfGNBP5 | -0.52 | 0.073 | -0.346 | 72.6778 | 73.85 | 73.85 | 9 | -6.7 |
| GMPP | | | | | 73.85 | 73.85 | 73.85 | | -6.2 |
| PGN | | | | | 67.55 | 67.55 | 67.55 | | -6.4 |
